# Can velmanase alfa be the next widespread potential therapy for alpha-mannosidosis?

**DOI:** 10.1097/JS9.0000000000000528

**Published:** 2023-06-21

**Authors:** Sundus Abdul Ghani, Sheeba Burney, Hassan ul Hussain, Maryam Abdul Wahid, Hassan Mumtaz

**Affiliations:** a Dow University of Health Sciences, Karachi; b Maroof International Hospital. Public Health Scholar, Health Services Academy, Islamabad, Pakistan

**Keywords:** alpha-mannosidosis, bone marrow transplant, lysosomal storage disorders, therapy

## Abstract

Alpha-mannosidosis (AM) is an autosomal recessive lysosomal storage disorder caused by reduced activity of the enzyme alpha-mannosidase. The disease is characterized by immunodeficiency, facial and skeletal abnormalities, impaired hearing, and intellectual disability. The clinical subtype of AM shows considerable variability in an individual, and at present, at least three clinical subtypes are suggested. Diagnosis is made by identification of deficiency of α-mannosidase activity in nucleated cells, like fibroblasts. The children are often born apparently normal as the disease is insidiously progressive, hence making early diagnosis essential. Along with supportive care, long-term therapeutic options include hematopoietic stem cell transplant, bone marrow transplantation, and enzyme replacement therapy. The possible benefits of these procedures must be weighed against the overall risk of procedure-related morbidity and mortality. Velmanase alfa is the first human recombinant form of alpha-mannosidase licensed and available for long-term enzyme replacement therapy. It is approved for treating non-neurologic manifestations of mild to moderate AM. The results obtained from different clinical trials provide evidence of the positive clinical effect of the recombinant enzyme on patients with AM. Different routes of diagnosis and unspecific initial symptoms of the disease lead to a delay in the initiation of treatment, resulting in accumulative morbidity. Thus, there is a dire necessity to create more awareness. Furthermore, additional multiple large-scale trials are needed to evaluate the long-term safety and efficacy of velmanase alfa.

## Background

Alpha-mannosidosis (AM) is a rare, autosomal recessive lysosomal storage disorder. AM is caused by mutations in the Mannosidase Alpha Class 2B Member 1 (MAN2B1) gene located on chromosome 19, which leads to lysosomal alpha-mannosidase deficiency. AM presents as a multisystemic disease with features mainly including immune deficiency with recurrent infections early in life, skeletal abnormalities like scoliosis, sternal deformities, hearing impairment along with gradual impairment in mental and motor functions^1^. The prevalence of AM is not exactly known; however, a study from Norway conducted by Malm D. *et al.* reported six patients out of a population of 4.5 million^2^. Other studies have shown a birth incidence of about 1:1 000 000 in the Netherlands, Portugal and Australia, and of 1:1 042 804 in the Czech Republic^3^.

Based on some recently published papers, three distinct clinical subtypes of AM have been suggested. Type 1 is the mild form, showing clinical symptoms after 10 years of age, without skeletal impairments and very slow progression; Type 2 is the moderate form, clinically presenting before the age of 10 years, with skeletal abnormalities, developmental of ataxia at age 20–30, and slow progression; Type 3 is the severe form, immediately recognized, with skeletal abnormalities and obvious progression, leading to an early death from CNS involvement or myopathy^1^.

The diagnosis of AM is confirmed by detecting low levels of acid alpha-mannosidase activity in peripheral blood leukocytes or other nucleated cells or it can also be confirmed with genetic testing via mutation analysis. Therefore, antenatal diagnosis is possible, via both biochemical and genetic testing. Genetic counseling can also be given to patients to explain the course and nature of the disease Additionally, elevated urinary secretion of mannose rich oligosaccharide is suggestive but not exactly diagnostic^1^.

## Conservative management

Other than supportive care, the only treatment option currently available is allogeneic hematopoietic stem cell transplant (HSCT) from a human leukocyte antigen matched donor, which has a variable outcome. The primary indication to offer HSCT in patients affected by AM is the preservation of neurocognitive function and the prevention of early death. However, patients who have received transplants are found to be at higher risk for autoimmune hemolytic anemia and pulmonary complications. The morbidity and mortality rate associated with HSCT must therefore be balanced^4^. Till date many papers have extensively described and focused entirely on the clinical manifestations and progression of AM, but different treatment options and their respective efficacies and adverse side effects still needs to be focused on. Thereupon, in our paper, we will be highlighting several potential therapeutic strategies for AM that have been proposed, that includes enzyme replacement therapy (ERT), bone marrow transplantation (BMT), and gene therapy.

## Bone marrow transplantation (BMT)

Another alternative method of treatment is BMT. It has been experimented for AM and has been shown to increase the chances of preventing cognitive decline and improving symptoms^5^.

## Correlation between phenotype, genotype, and mutant MAN2B1 subcellular localization

A study conducted by Borgwardt *et al.* ^6^ in the past identified a potential relationship between three *MAN2B1* genotype/subcellular localization subgroups (G1, G2, and G3) and AM severity. However, there is a lack of sufficient data on this. A recent study suggested that antidrug antibodies have a limited effect on the clinical benefit of Velmanase Alpha in AM patients regardless of the *MAN2B1* subgroup^7^. ERT for AM: Velmanase alpha is an intravenously (IV) administered recombinant human lysosomal alpha-mannosidase, approved by Food and Drug Administration on 16th February 2023 to be used as an ERT for AM^8^.

### Dosage and pharmacokinetics

IV Velmanase alpha administration 1 mg/kg once weekly was found to be well tolerated^9^. The half-life of Velmanase alpha increased with each dose and it was found that the increased half-life was not influenced by the accumulation of the drug due to repeated infusions^8^.

### Efficacy and side effects of IV velmanase

Different randomized controlled trials showed that there was a decrease in oligosaccharide levels in the serum, urine, and cerebrospinal fluid while there was an improvement in the 3-Minutes-StairClimb-Test (3MSCT), 6-Minutes-Walk-test (6MWT), and cognitive function in the Leiter *R* test^8-10^. It also showed promising results in the patients who were given therapy early in the disease course^9^. Alongside this beneficial potential, mild infusion-related reactions were observed in 2 out of 10 patients, which included chills, temperature rise, and headaches^8^. Antidrug antibodies developed in 4 out of 5 patients and 2 out of 5 patients experienced infusion-related reactions, which resolved without withdrawal from the study^10^. The nonserious adverse events included mild infections, muscle and skeletal pain, headache, and elective surgery. These adverse events were mild to moderate in severity and were not related to Velmanase alpha^8^. A detailed comparison of the efficacy and side effects of Velmanase alpha is provided in Table 1.

**Table 1:** Comparison of the efficacy and side effects of Velmanase alpha in different RCTs.

First author	Phase of trial	ClinicalTrial.gov identifier	Efficacy	Adverse effects
Borgwardt L^8^	½	NCT01268358 NCT01681940	S-oligo decreased 88.6% U-oligo decreased 54.1% CSF-oligo decreased 25.7% Improvements in 3MSCT (31 steps) Improvements in 6MWT (60.4 m) Cognitive function improved over 4 months (0.34) in Leiter R test.	No AEs related to the drug were observed to stop the infusion permanently.
Borgwardt L^9^	3	NCT01681953	S-oligo decreased 62.9% 3MSCT improved by 3.9%.	No treatment emergent AEs were reported to stop the infusion permanently.
Guffon N^10^	2	NCT02998879	S-oligo decreased 32-83% from baseline Improvements in 3MSCT (28.5 steps).	Vomiting (100%) Pyrexia (80%) Cough (80%) Otitis media (80%) Nasopharyngitis (60%) Rhinitis (60%) Diarrhea (60%) Anal pruritus (20%).

3-Minutes-StairClimb-Test (3MSCT); 6-Minutes-Walk-test (6MWT); AEs, Adverse events; CSF-oligo, CSF oligosaccharide; S-oligo, Serum oligosaccharide; U-oligo, Urine oligosaccharide.

## Future prospects and recommendations

### Importance of an early diagnosis

Diagnosis takes months to years due to the different routes and complexity of the diagnostic pathway. Initial symptoms of AM are often nonspecific and thus are managed by multiple healthcare professionals rather than being considered as part of a multisystemic disease. This leads to a delay in diagnosis, which results in accumulative morbidity. Therefore, there is a dire need for greater awareness of AM amongst healthcare professionals and more support for patients and caregivers, especially those living in rural areas. Increased awareness of AM may lead to a shorter time in reaching its diagnosis. Data from a survey has shown that early diagnosis not only improves symptoms but also the patient’s quality of life^11^.

Furthermore, newborn screening can be essential in identifying affected individuals before the onset of severe irreversible pathology.

Drug usage for patients below 6 years of age is still under investigation as the rarity and complexity of diagnosis lead to diagnostic delay. There is no published data; therefore, more research is needed to stimulate the effects of ERT in young patients below 6 years of age^12^. Furthermore, there is a need to determine the long-term safety and efficacy of BMT therapy. Additionally, future studies are required to assess the correlation between *MAN2B1* subgroups and ADA development to provide meaningful insight into the disease management of AM patients.

### The SPARKLE registry

The SPARKLE registry is a designed multicenter, multinational, noninterventional, prospective cohort study to provide real-world data on European patients, which started enrollment of patients with AM in 2020. Patients in this cohort will be followed for up to 15 years. It will increase the understanding of this disease’s natural course, clinical manifestations, and progression in treated and untreated patients, specifically on the long-term safety and effectiveness of velmanase alfa treatment^13^. Hence, we suggest further multiple large-scale trials, longer-term follow-up of patients, and the future placebo-controlled phase 3 trial to provide more valuable support to velmanase alfa’s long-term safety and efficacy.

## Ethics approval and consent to participate

Not applicable.

## Consent for publication

Not applicable.

## Sources of funding

None.

## Author contribution

S.A.G.: conceptualization, data curation, investigation, visualization, writing—original draft, S.B.: conceptualization, data curation, resources, writing—original draft; H.u.H.: conceptualization, investigation, resources, visualization, writing— original draft, review and editing; M.A.W.: data curation, writing—original draft; H.M.: review and editing. All authors have read and approved the final version of the manuscript. The corresponding author had full access to all of the data in this study and takes complete responsibility for the integrity of the data.

## Conflicts of interest disclosure

The authors declare no conflicts of interest.

## Research registration unique identifying number (UIN)

1. Name of the registry: Not applicable.
2. Unique Identifying number or registration ID: Not applicable.
3. Hyperlink to your specific registration (must be publicly accessible and will be checked): Not applicable.

## Guarantor

Hassan Mumtaz.

## Availability of data and materials

Any of the authors can be contacted for data and materials.

## Data availability statement

Data will be provided by the authors upon request.

## Provenance and peer review

Paper was not invited.

## Assistance with the study

None.

## Presentation

Not applicable.
